# ﻿Molecular and morphological characterization of four new *Phyllosticta* species (Botryosphaeriales, Phyllostictaceae): Genomic insights into evolutionary dynamics and metabolic adaptation

**DOI:** 10.3897/imafungus.16.168055

**Published:** 2025-10-20

**Authors:** Meng-Yuan Zhang, Zhao-Xue Zhang, Ya-Ling Wang, Xiu-Guo Zhang, Zhuang Li

**Affiliations:** 1 Shandong Provincial Key Laboratory for Biology of Vegetable Diseases and Insect Pests, College of Plant Protection, Shandong Agricultural University, Taian, 271018, China Shandong Agricultural University Taian China

**Keywords:** *
Botryosphaeriales
*, metabolic adaptation, phylogenomics, species complexes, taxa

## Abstract

The genus *Phyllosticta* comprises diverse plant-associated fungi, many of which are significant pathogens or endophytes with complex taxonomic histories. Traditional classification, reliant on morphology and host associations, has long been challenged by overlapping traits, necessitating integrative approaches combining molecular and phenotypic data. In this study, four new *Phyllosticta* species—*P. decaspermi***sp. nov.** (from *Decaspermum montanum* and dead leaves), *P. morellae***sp. nov.** (from *Morella rubra*), *P. clematidea***sp. nov.** (from *Clematis vitalba*), and *P. pittosporicola***sp. nov.** (from *Pittosporum illicioides* and dead leaves)—are described based on multi-locus phylogenetic analysis (ITS, LSU, *tef1*, *act*, and *gpdh*) and morphological characterization. These taxa are assigned to the *P. capitalensis* and *P. concentrica* species complexes, expanding the known diversity of these clades. Phylogenomic analysis using 5,399 orthologous protein clusters confirmed robust species boundaries within *Phyllosticta*, mirroring patterns observed in other fungal groups where phylogenomics resolves ambiguous relationships. Gene family analysis revealed a high proportion of conserved single-copy orthologs, indicating stable core functions, alongside unique gene families likely underlying ecological specialization. KEGG metabolic pathway analysis highlighted species-specific adaptations: for example, enhanced “Protein processing in endoplasmic reticulum” in saprophytic species and active “Amino sugar and nucleotide sugar metabolism” in host-associated taxa, reflecting functional divergence linked to lifestyle. These findings align with broader fungal evolutionary trends, where nuclear genomic divergence and potential mitochondrial contributions (e.g., intron dynamics) shape metabolic strategies enabling adaptation to diverse hosts. This study enhances our understanding of *Phyllosticta* taxonomy and evolution, emphasizing the utility of integrative approaches in resolving fungal diversity.

## ﻿Introduction

*Phyllosticta* Pers., established by Persoon in 1818, is a well-documented genus with a complex taxonomic history. Its type species, initially designated as *Phyllosticta convallariae* (Donk 1968), was later synonymized with *P. cruenta* (van der Aa 1973; Wikee et al. 2013a). Ecologically, *Phyllosticta* is prominent as a plant pathogen, causing leaf spots and fruit diseases across a wide range of economically valuable crops and ornamentals. Notable examples include *P. citricarpa* (responsible for citrus black spot; Baayen et al. 2002; Glienke et al. 2011), *P. ampelicida* (causal agent of grape black rot in North America; Kuo and Hoch 2018), and *P. ophiopogonis* (inducing leaf spots on *Ophiopogon japonicus* in Thailand; Wikee et al. 2012). Additionally, some species such as *P. capitalensis* exhibit dual ecological roles, acting as common endophytes with antagonistic activity against pathogens like *P. citricarpa* and utilizing melanized appressoria for host penetration (Wikee et al. 2013b; Tran et al. 2019). To date, the Index Fungorum (accessed 6O ctober 2025) lists 3,228 names under *Phyllosticta*, highlighting its taxonomic diversity.

The taxonomic placement of *Phyllosticta* has undergone significant revisions over time. Seaver (1922) first classified it within the family *Phyllostictaceae* Fr. and order *Phyllostictales*, but subsequent studies reclassified it into *Botryosphaeriaceae* (Theiss. & Syd.) under *Botryosphaeriales* (C.L. Schoch et al.) (Crous et al. 2006; Schoch et al. 2006; Liu et al. 2012). Phylogenetic analyses by Wikee et al. (2013a) and Phillips et al. (2019) later positioned *Phyllosticta* as a sister clade to *Botryosphaeriaceae*, leading to its reclassification back to *Phyllostictaceae* within *Botryosphaeriales* (Zhang et al. 2023). In recent years, integrative approaches combining molecular data and morphological traits have accelerated the description of new species (e.g., Su and Cai 2012; Wang et al. 2012, 2013; Wong et al. 2012; Zhang et al. 2012, 2013; Wikee et al. 2013a; Wulandari et al. 2013; Crous et al. 2014–2021; Zhou et al. 2015; Guarnaccia et al. 2017; Lin et al. 2017; Hattori et al. 2020; Norphanphoun et al. 2020). Norphanphoun et al. (2020) further refined this diversity by proposing six species complexes (*P. capitalensis*, *P. concentrica*, *P. cruenta*, *P. owaniana*, *P. rhodorae*, and *P. vaccinii*) based on a five-locus dataset, with additional new taxa or records emerging since then (Zhang et al. 2022; Jiang et al. (2023); Gomdola et al. 2024). Morphologically, *Phyllosticta* species exhibit distinct sexual and asexual traits, though overlap complicates species delimitation. Sexual morphs feature erumpent, uniloculate ascomata with pseudoparaphyses, clavate to ellipsoidal asci, and aseptate, hyaline ascospores often with mucoid caps (van der Aa 1973; Wong et al. 2012; Wikee et al. 2013a). Asexual morphs produce hyaline, aseptate conidia with a mucilaginous sheath and variable apical appendages (Wikee et al. 2013a), while spermatia are cylindrical to dumbbell-shaped with terminal guttules. Due to such morphological overlap, multi-locus phylogenetic analysis is critical for species delimitation (Norphanphoun et al. 2020).

With advances in sequencing, genomics has become a powerful tool for phylogenetic inference and understanding pathogenic mechanisms (Manamgoda et al. 2011; Schoch et al. 2012; Jeewon et al. 2013; David et al. 2016; Mesny et al. 2021; Tsers et al. 2023). However, genomic resources for *Phyllosticta* remain limited; as of 6 October 2025, only seven species have publicly available genomes on NCBI. This study aims to address this gap by exploring *Phyllosticta* diversity, describing four new species using molecular phylogenetics and morphology, and conducting genome/transcriptome sequencing of these new taxa. By comparing these data with existing genomic information, we seek to clarify genetic relationships and functional differences, enhancing our understanding of the biology and evolutionary history of these novel species.

## ﻿Materials and methods

### ﻿Isolation and morphological studies

From 2023 to 2024, dead, healthy, and diseased leaves of specific host plants—*Clematis vitalba*, *Decaspermum montanum*, *Pittosporum illicioides*, and *Morella rubra*—were collected in Hainan and Yunnan provinces. Thirty-two specimens were transported to the laboratory in paper bags for subsequent fungal isolation and analysis. Fungal isolates were obtained via the tissue isolation method (Zhang et al. 2023, 2025). For each sample, 5 × 5 mm leaf lesion fragments were excised from the margins of symptomatic tissues. The surface-sterilization process involved sequential steps: immersion in 75% ethanol for 30 seconds, rinsing in sterile distilled water, treatment with 5% sodium hypochlorite solution for 30 seconds, and a final rinse in sterile distilled water for 1 minute (Jiang et al. 2021). After drying on sterilized tissue paper, the fragments were placed on 2% potato dextrose agar (PDA) and incubated at 25 °C for 2–4 days. Actively growing hyphal tips were transferred to fresh PDA plates to culture pure strains for morphological examination. Colony morphology was documented on days 7 and 14 using a Canon Powershot G7X digital camera (Canon Co., Ltd, Beijing, China). Micromorphological features were observed with an Olympus SZX10 stereomicroscope and an Olympus BX53 microscope (Olympus Corporation, Tokyo, Japan), both equipped with an Olympus DP80 high-resolution color digital camera (Olympus Corporation, Tokyo, Japan) for imaging fungal structures. All fungal strains were preserved in 10% sterilized glycerin at 4 °C for future studies. For morphological quantification, 30 measurements were taken for each structural feature using Digimizer software (https://www.digimizer.com/). Holotype specimens were deposited in the Herbarium of Plant Pathology, Shandong Agricultural University (HSAUP). Ex-type and other living cultures were deposited at the China General Microbiological Culture Collection Center (CGMCC, Beijing, China) and the Shandong Agricultural University Culture Collection (SAUCC, Shandong, China). Taxonomic information for newly described taxa was registered in MycoBank (http://www.mycobank.org).

### ﻿DNA extraction and sequencing

Genomic DNA was extracted from fungal mycelia cultured on PDA using either a modified cetyltrimethylammonium bromide (CTAB) protocol or a commercial kit (OGPLF-400, GeneOnBio Corporation, Changchun, China), following methods optimized for Sanger sequencing (Wang et al. 2023; Zhang et al. 2023, 2025). For multi-locus phylogenetic analysis, five genetic regions were amplified and sequenced using eight primer pairs: the internal transcribed spacer (ITS) region including the 5.8S rRNA gene (primers ITS5/ITS4; White et al. 1990), the large subunit (LSU) of the rRNA gene (primers LR0R/LR5; White et al. 1990), the translation elongation factor 1-alpha gene (*tef1*; primers EF1-728F/EF2; O’Donnell et al. 1998; Carbone and Kohn 1999), the actin gene (*act*; primers ACT-512F/ACT-783R; Carbone and Kohn 1999), and the glyceraldehyde-3-phosphate dehydrogenase gene (*gpdh*; primers GDF1/GAPDH; Myllys et al. 2002). PCR amplifications were performed in 20 μL reaction volumes containing 10 μL 2 × Hieff Canace® Plus PCR Master Mix (with dye; Yeasen Biotechnology, Cat No. 10154ES03), 0.5 μL each of forward and reverse primers (10 μM; TsingKe, Qingdao, China), 1 μL template genomic DNA, and distilled deionized water to adjust the final volume. Amplicons were visualized on 2% agarose gels and purified using a Gel Extraction Kit (AE0101-C, Shandong Sparkjade Biotechnology Co., Ltd.). Bidirectional Sanger sequencing was conducted on an Eppendorf Master Thermocycler (Hamburg, Germany) at Youkang Biotechnology Co., Ltd (Qingdao, China). All newly generated sequences were deposited in GenBank (Suppl. material 1).

### ﻿Library construction, quality control and whole-genome sequencing

Library preparation and high-throughput sequencing were performed by Novogene Co., Ltd. (Beijing, China). FASTQ-formatted sequencing data were generated, which contained both sequence reads and their corresponding sequencing quality scores (Cock et al. 2010). Raw sequencing data derived from the sequencing platform were preprocessed using fastp (https://github.com/OpenGene/fastp) to yield high-quality clean data for downstream analyses (Chen et al. 2018). All clean data were deposited in the National Center for Biotechnology Information (NCBI) database under BioProject accession number PRJNA1302140, while the GenBank accession numbers corresponding to the genome sequences are provided in Table 1.

**Table 1.** BioSample and SRA NCBI number of the taxa used in phylogenomic analyses in this study.

Species	Strains	BioSample	SRA NCBI*	References
*Pestalotiopsis fici*	W106-1	SAMN02369365		Yu et al. (2023)
*Phyllosticta ampelicida*	Galicia	SAMN20693606		
*P. capitalensis*	CBS 128856	SAMN05877936	SRS2357592	
	**CGMCC3.28668**	**SAMN50463590**	**SRR34983904**	**This study**
*P. citricarpa*	CBS 122482	SAMN16773465	SRS7805235	
*P. citriasiana*	CBS 120426	SAMN16773552	SRS7805223	
*P. citrichinensis*	CBS 129764	SAMN16773304	SRS7805227	Buijs et al. 2022
*P. citribraziliensis*	CPC 17464	SAMN18252025		
***P. clematidea***	**CGMCC3.28671**	**SAMN50463589**	**SRR34983905**	**This study**
***P. decaspermi***	**CGMCC3.28667**	**SAMN50463587**	**SRR34983907**	**This study**
***P. elliptica***	**CGMCC3.28672**	**SAMN50463591**	**SRR34983903**	**This study**
***P. fujianensis***	**SAUCC 1366-3**	**SAMN50463584**	**SRR34983910**	**This study**
***P. morellae***	**CGMCC3.28669**	**SAMN50463588**	**SRR34983906**	**This study**
*P. paracitricarpa*	CBS 141358	SAMN17676028	SRS8243309	
***P. rhododendri***	**CGMCC3.28673**	**SAMN50463592**	**SRR34983902**	**This study**
***P. saprophytica***	**SAUCC1516-2**	**SAMN50463585**	**SRR34983909**	**This study**
***P. turpiniae***	**SAUCC2864-3**	**SAMN50463586**	**SRR34983908**	**This study**
***P. wuzhishanensis***	**CGMCC3.28670**	**SAMN50463593**	**SRR34983901**	**This study**

*Species information described in this study is marked in bold.

### ﻿Genome assembly and annotation

Genomic data were assembled using SPAdes v3.12.0 (Bankevich et al. 2012). Genome annotation encompassed three main steps: i) masking of repetitive sequences (RepeatMasker v4.1.4; RepeatModeler v2.0.3, https://www.repeatmasker.org/); ii) annotation of non-coding RNAs (RNAmmer v1.2; tRNAscan-SE v2.0); and iii) annotation of gene structures, which included RNA-seq-based prediction (Trinity v2.14.0, HISAT2 v2.2.1, StringTie v2.2.0), ab initio prediction (BRAKER2), and homology-based protein prediction (GeMoMa v1.9) (Grabherr et al. 2011; Pertea et al. 2015; Keilwagen et al. 2016, 2018; Bruna et al. 2021). The final genome and annotation files were integrated using EVM and PASA (Haas et al. 2008, 2011).

### ﻿Phylogenetic and phylogenomic analysis

Consensus sequences generated in this study were subjected to BLAST searches against NCBI’s GenBank nucleotide database to identify closely related reference sequences (Zhang et al. 2000). For multi-locus phylogenetic analysis, we used a backbone alignment from Jiang et al. (2023) and integrated newly generated sequences (Suppl. material 1) with related GenBank-retrieved sequences. Alignments of the five loci (ITS, LSU, *tef1*, *act*, and *gpdh*) were performed using MAFFT 7 (Katoh et al. 2019) and manually corrected in BioEdit (Hall 2006). To clarify isolate identities, phylogenetic analyses were conducted for each locus individually, followed by a concatenated analysis of the combined dataset (ITS-LSU-*tef1*-*act*-*gpdh*). Phylogenetic inference followed the methods of Zhang et al. (2023, 2025), employing both Maximum Likelihood (ML) and Bayesian Inference (BI) algorithms. ML analysis was run either via the CIPRES Science Gateway using RAxML-HPC2 on ACCESSv. 8.2.12 (Miller et al. 2012) or RAxML-NG v1.2.1, using default parameters with 1,000 rapid bootstrap replicates under the GTR nucleotide substitution model. This model was chosen based on prior model selection analyses using ModelFinder (Kalyaanamoorthy et al. 2017) implemented in IQ-tree, which evaluates model fit via the Bayesian information criterion (BIC). BI analysis was performed in MrBayes v3.2.7a with a fast bootstrap algorithm and automatic stopping criteria (Zhang et al. 2023); the burn-in fraction was set to 0.25, and posterior probabilities (PP) were calculated from remaining trees.

For phylogenomic analysis, the final annotated data were processed to retain the coding protein genes and the longest transcript. OrthoFinder v2.5.5 was used to perform gene family analysis and identify 6364 clusters of orthologous proteins (COPs) (Emms and Kelly 2015, 2019). Multiple sequence alignments of these COPs were generated with ParaAT v1.0, and resulting sequences were concatenated into a supergene using seqkit v2.7.0 (Zhang et al. 2012; Shen et al. 2016). Phylogenomic trees were constructed following Zhang et al. (2025) using RAxML-NG v1.2.1 under the GTR+G+I model with 1000 bootstrap replications. All resulting trees were visualized using FigTree v1.4.4 or ITOL (Interactive Tree of Life, accessed 6 October 2025; Letunic and Bork 2021), with final layout adjustments made in Adobe Illustrator CC 2019.

### ﻿Kyoto Encyclopedia of Genes and Genomes (KEGG) metabolic pathways annotations

Gene models were predicted using genome sequences and corresponding annotation files. For each gene, the longest protein sequence was extracted using TransDecoder v5.7.1 (https://github.com/TransDecoder) to facilitate downstream functional analysis.

Functional annotation of these protein sequences was performed with eggNOG-mapper v5.0.2 (Huerta-Cepas et al. 2019; https://github.com/eggnogdb/eggnog-mapper). The longest protein sequences were mapped to orthologous groups in the eggNOG database using the emapper.py tool, which employs the Diamond algorithm for rapid sequence alignment (Buchfink et al. 2021). The annotation process was executed with specific parameters: a seed ortholog e-value threshold of 1e-5, with the taxonomic scope restricted to *Ascomycota* to ensure relevance. This annotation workflow yielded detailed functional classifications, including assignments to KEGG metabolic pathways, providing insights into the metabolic and functional potential of the predicted genes.

## ﻿Results

### ﻿Phylogenetic analysis

**Figure 1.**
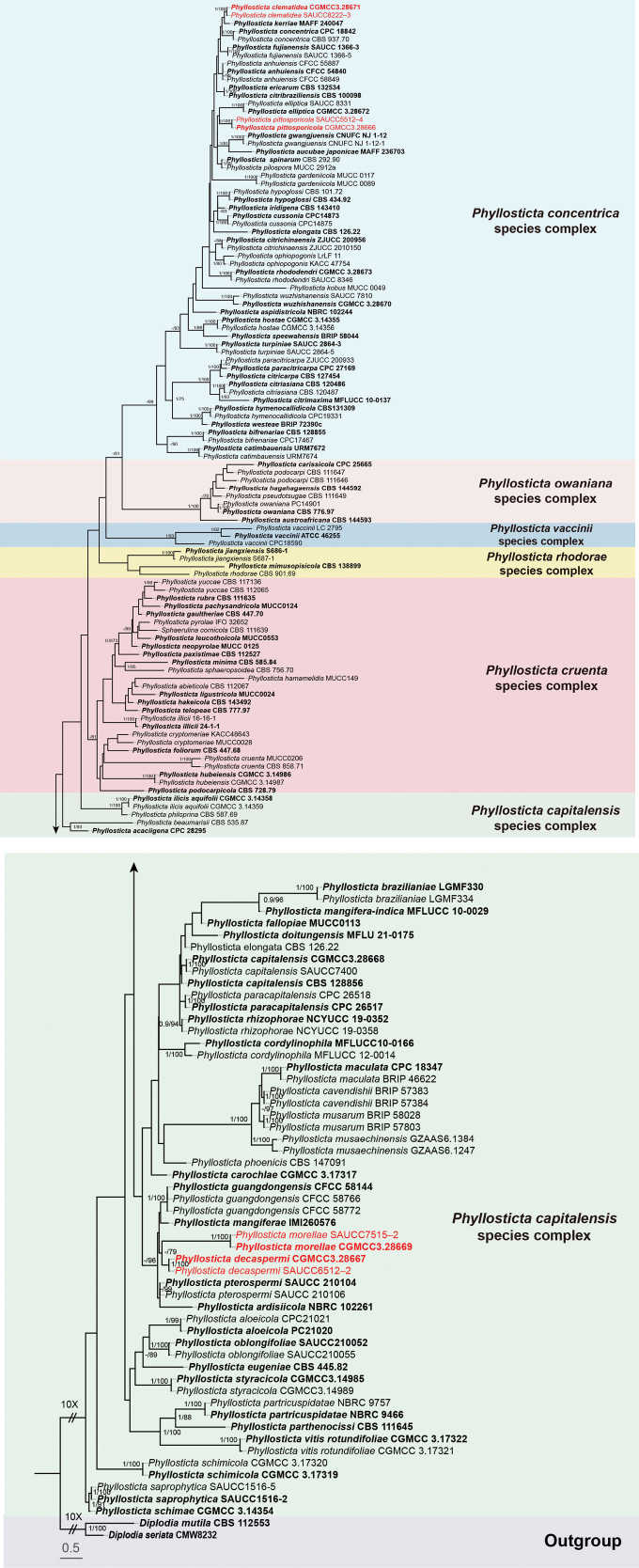
Phylogram of the *Phyllosticta*, inferred from a concatenated alignment of ITS, LSU, *tef1*, *act*, and *gpdh* sequences. *Diplodia mutila* (CBS 112553) and *D. seriata* (CMW8232)were used as outgroup taxa. BI posterior probabilities and ML bootstrap support values above 0.90 and 70% are shown at the first and second position, respectively. Ex-type cultures are highlighted in bold, while strains obtained in this study are marked in red. Some branches have been shortened for layout optimization, indicated by double diagonal lines with the corresponding reduction factor. The scale bar at the bottom left represents the number of substitutions per site.

A total of 159 isolates representing the *Phyllosticta* species were subjected to phylogenetic analysis, with *Diplodia mutila* (CBS 112553) and *D. seriata* (CMW8232) designated as outgroup taxa. The final alignment consisted of 2,911 characters, corresponding to the following loci: 1–656 (ITS), 657–1,401 (LSU), 1,402–1,906 (*tef1*), 1,907–2,173 (*act*), and 2,174–2,911 (*gpdh*). Among these, 1,721characters were constant, 209 were variable and parsimony-uninformative, and 981 were parsimony-informative. ML analysis yielded the best-scoring RAxML tree with a final likelihood value of –32,983.584450. The alignment contained 1,480 distinct patterns, with 34.56% undetermined characters or gaps. The estimated base frequencies were as follows: A = 0.201106, C = 0.315313, G = 0.263647, T = 0.219934; substitution rates AC = 1.327702, AG = 3.656578, AT = 1.618872, CG = 1.168950, CT = 6.497707, GT = 1.0. The gamma distribution shape parameter alpha was estimated at0.589727. As the ML and BI analyses produced topologically congruent trees, only ML tree (Fig. 1) is presented, with posterior probabilities and bootstrap provided for well-supported clades. Based on the five-gene phylogenetic framework (Fig. 1), the 159 isolates were assigned to 103 species. The present study identified three novel species, viz. *P. clematidea* sp. nov., *P. decaspermi* sp. nov., *P. pittosporicola* sp. nov., and *P. morellae* sp. nov.

### ﻿Phylogenomic analysis in *Phyllosticta*

**Figure 2.**
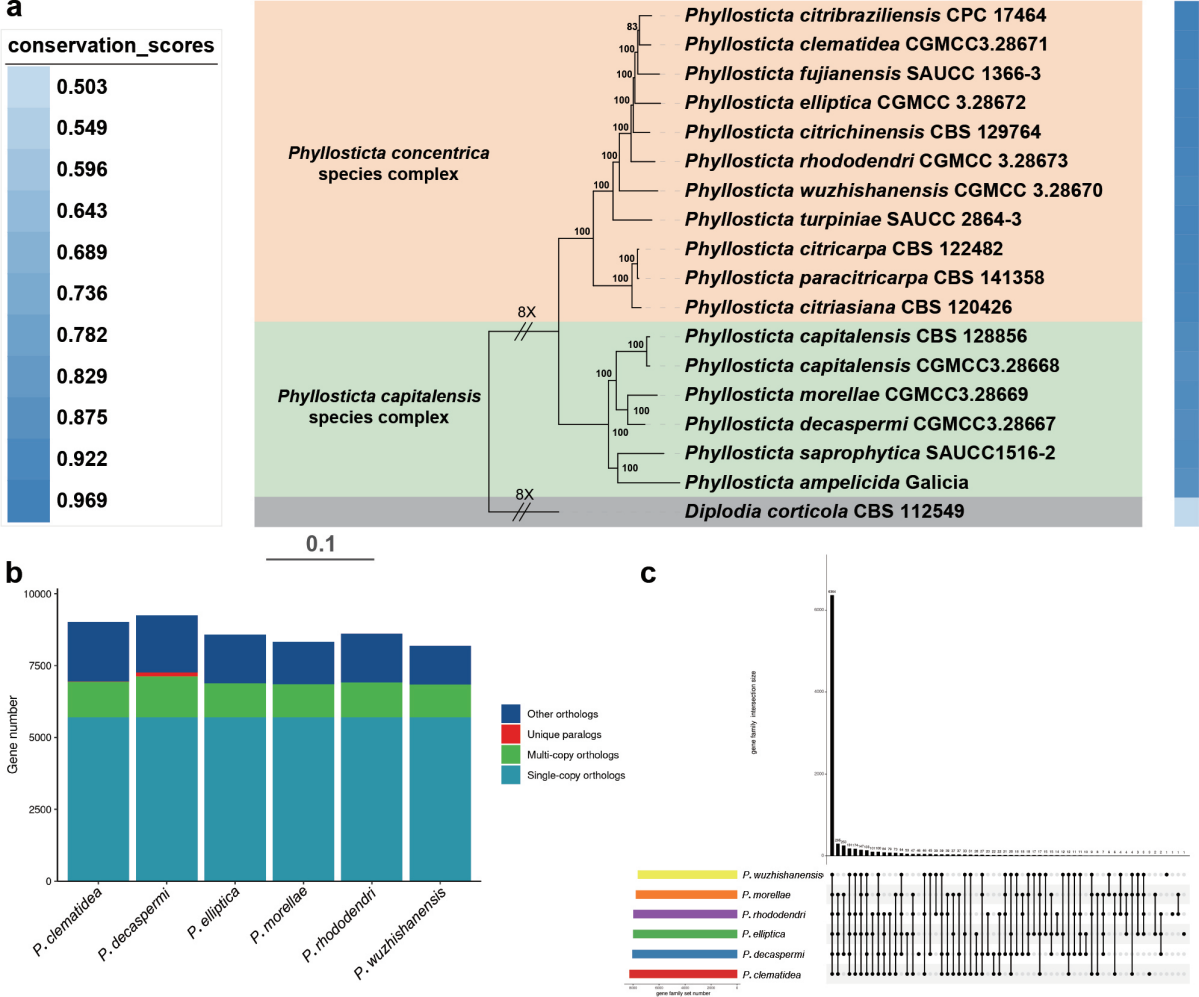
Gene family analysis within *Phyllosticta*. **a** A Maximum Likelihood phylogenomic tree illustrating relationships within *Phyllosticta* based on 5,399 clusters of orthologous proteins. Maximum likelihood bootstrap values (≥ 50%) are indicated along branches.Complexes are highlighted in different colors. First layer: sequence conservation (light blue to blue). The scale bar at the lower right represents substitutions per site **b** Bar chart of homologous genes for each strain **c** UpSet plot of six strains, showing the intersection counts betweendifferent strains in the form of a bar graph.

In this study, we sequenced the genomes of 10 isolates within *Phyllosticta* and included published genomes of 7 additional species from NCBI (https://www.ncbi.nlm.nih.gov/datasets/). *Diplodia corticola* (CBS 112549) served as the outgroup taxon. The Maximum Likelihood (ML) tree was constructed from 5,399 clusters of orthologous proteins (Fig. 2).

From the phylogenomic tree (Fig. 2a), it is evident that different *Phyllosticta* species form distinct clusters, such as the *Phyllosticta concentrica* species complex and the *Phyllosticta capitalensis* species complex. The bootstrap values (≥ 50%) along the branches reflect the support strength for the phylogenetic relationships. The sequence conservation gradient (from light blue to dark blue) reveals evolutionary differences in gene families among various species. Species with high conservation are likely to be more similar in genes related to core metabolism or basic life activities.

In the bar chart of gene family analysis (Fig. 2b), there are both commonalities and differences in the composition of homologous genes among strains. Single-copy orthologous genes account for a relatively large proportion, indicating that these species have many conserved single-gene functions in the basic genetic framework. The presence of unique genes may confer specific ecological adaptation or functional differentiation potential to each strain. For example, they may show specificity in host interaction and environmental stress response.

The UpSet plot (Fig. 2c) intuitively presents the intersection of gene families among six strains. It helps to identify the gene family modules shared by different strains and the unique gene families. The shared modules may be associated with the core biological functions of the *Phyllosticta*, such as genes related to basic metabolic pathways. The unique gene families of strains may be acquired during long-term evolution to adapt to specific ecological niches (such as different host plants and environmental conditions). For instance, some genes are involved in host pathogenicity and secondary metabolite synthesis. These differences provide genetic-level clues for deeply understanding the ecological adaptability and functional differentiation of *Phyllosticta* species. Overall, the gene family analysis reveals the evolutionary relationships and functional differentiation patterns among *Phyllosticta* species from the dimensions of phylogeny, gene composition, and intersection, laying a foundation for subsequent exploration of their biological characteristics and ecological functions.

### ﻿KEGG metabolic pathway analysis in *Phyllosticta*

**Figure 3.**
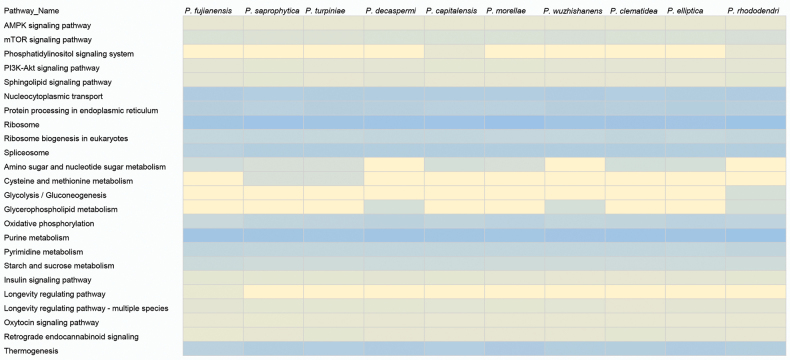
KEGG Pathway Heatmap of Various Species. This heatmap visualizes the relative activity levels of selected metabolic pathways across different species, as indicated by the color gradient from yellow (lower activity) to blue (higher activity).

In this study, genomes of ten species, namely *Phyllosticta fujianensis*, *P. saprophytica*, *P. turpiniae*, *P. decaspermi*, *P. capitalensis*, *P. morellae*, *P. wuzhishanensis*, *P. clematidea*, *P. elliptica*, and *P. rhododendri*, were annotated. Then, KEGG pathway heatmap analysis was performed on them. The color gradient in the heatmap ranges from yellow, representing lower pathway activity, to blue, indicating higher activity. This visual representation allows for a straightforward comparison of metabolic pathway activities across these species (Fig. 3).

Take *P. fujianensis*, *P. saprophytica*, and *P. rhododendri* for in-depth analysis. For *P. fujianensis*, the “Cysteine and methionine metabolism” pathway shows a yellow hue, implying relatively low activity in sulfur-containing amino acid metabolism. In contrast, *P. saprophytica* has a blue shade in “Protein processing in endoplasmic reticulum”, reflecting vigorous protein folding and modification processes, which may be crucial for its secretion of extracellular enzymes to decompose organic matter in the saprophytic lifestyle.

*Phyllosticta rhododendri* stands out with a blue color in “Amino sugar and nucleotide sugar metabolism”, suggesting active biosynthesis of cell wall components like chitin, potentially helping it establish interactions with host plants. These distinct pathway activities indicate that *P. fujianensis* may prioritize basic metabolic conservation in amino acid processes, *P. saprophytica* invests in protein quality control for saprophytic nutrition acquisition, and *P. rhododendri* focuses on cell wall-related sugar metabolism for ecological adaptation, showcasing species-specific metabolic strategies shaped by evolutionary and environmental pressures.

### ﻿Taxonomy

#### ﻿*Phyllosticta capitalensis* species complex

Based on molecular analysis and morphological characteristics, the *Phyllosticta capitalensis* species complex comprises 34 species: *P. acaciigena*, *P. aloicola*, *P. ardisiicola*, *P. beaumarisii*, *P. brasiliana*, *P. capitalensis*, *P. carochlae*, *P. cavendishii*, *P. cordylinophila*, *P. decaspermi*, *P. doitungensis*, *P. eugeniae*, *P. fallopiae*, *P. guangdongensis*, *P. ilicis-aquifolii*, *P. maculata*, *P. mangiferae*, *P. mangifera-indicae*, *P. morellae*, *P. musaechinensis*, *P. musarum*, *P. oblongifoliae*, *P. paracapitalensis*, *P. parthenocissi*, *P. partricuspidatae*, *P. philoprina*, *P. phoenicis*, *P. pterospermi*, *P. rhizophorae*, *P. saprophytica*, *P. schimae*, *P. schimicola*, *P. styracicola*, and *P. vitis-rotundifoliae*.

##### ﻿ Phyllosticta decaspermi

Taxon classificationAnimaliaBotryosphaerialesPhyllostictaceae

M.Y. Zhang, Z.X. Zhang & X.G. Zhang sp. nov.

929DB801-5E90-5D3A-A1BA-65A02D6C83A6

857224

###### Etymology.

The specific epithet “*decaspermi*” refers to the host plant *Decaspermum montanum*.

###### Type.

CHINA • Hainan Province, Hainan Diaoluoshan National Forest Park, on diseased leaves of *Decaspermum montanum* Ridl., 27March 2023, M.Y. Zhang (holotype HSAUP6466–1), ex-type living culture CGMCC3.28667.

###### Description.

**Figure 4.**
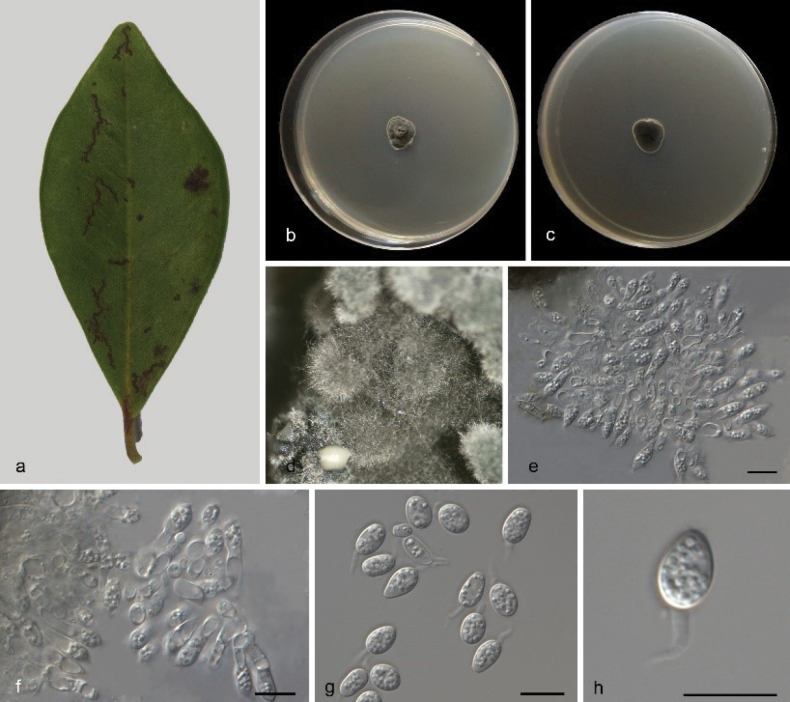
*Phyllosticta decaspermi* (CGMCC3.28667) **a** diseased leaf of *Decaspermum montanum***b, c** colonies (left-above, right-reverse) after 7 d on PDA**d** conidiomata **e, f** conidiogenous cells with conidia **g, h** conidia. Scale bars: 10 μm (**e–h**).

Leaf endogenic and associated with leaves of ***Decaspermum montanum*** Ridl. Asexual morph: ***Conidiomata*** pycnidial, mostly aggregated in clusters, black, erumpent. In PDA culture exuding colourless to opaque conidial masses within 12 days or longer. ***Conidiophores*** indistinct, often reduced to conidiogenous cells. ***Conidiogenous cells*** 6.5–15.5 × 2–4 μm, subcylindrical, ampulliform, hyaline, smooth. ***Conidia*** 10–13.5 × 5.5–7 μm, hyaline, aseptate, thin and smooth walled, coarsely guttulate, ovoid, ampulliform, ellipsoidal to subglobose, enclosed in a thin mucoid sheath. ***Sheath*** 1–2.5 μm thick and bearing a hyaline, apical mucoid appendage. ***Appendages*** 3–7 × 0.5–1 μm, flexible, unbranched, tapering towards an acutely rounded tip. Sexual morph not observed, see Fig. 4.

###### Culture characteristics.

Colonies on PDA 12–16 mm in diameter after 7 d at 25 °C in darkness, with a growth rate of 1.7–2.3 mm/day, white and undulate at edge, grey-green in center in obverse and reverse.

###### Additional specimen examined.

CHINA • Hainan Province, Hainan Diaoluoshan National Forest Park, on dead leaves, 27 March 2023, M.Y. Zhang (HSAUP6512–2), living culture SAUCC6512–2.

###### Notes.

Two isolates from leaf spots of *Decaspermum montanum* phylogenetically clustered into a well-supported clade (1.00/100), which is closely related to *Phyllosticta guangdongensis* (CFCC 58144), *P. mangiferae* (IMI260.576) and *P. morellae* (CGMCC3.28669). However, *P. decaspermi* differs from *P. guangdongensis* by 68 nucleotides (10/480 in ITS, 5/740 in LSU, 38/199 in *tef1*, 6/195in *act*, and 9/594 in *gpdh*), from *P. mangiferae* by 75 nucleotides (8/518 in ITS, 4/740 in LSU, 36/198 in *tef1*, 6/195 in *act*, and 21/615 in *gpdh*) and from *P. morellae* by 114 nucleotides (6/559 in ITS, 7/743 in LSU, 84/324 in *tef1*, 5/196 in *act*, and 12/715 in *gpdh*). In morphology, they are distinguished by different hosts (*Decaspermum montanum* vs. *Viburnum odoratissimum* vs. *Mangifera indica* vs. *Morella rubra*) and narrower conidia in *P. decaspermi* than *P. guangdongensis*, *P. mangiferae* and *P. morellae* (10–13.5 × 5.5–7 μm vs. 10–14 × 6–8 μm vs. 10.0–12.0 × 6.0–7.0 μm vs. 8.4–13.7 × 5.7–9.2 μm) (Jeewon and Hyde 2016; Wang et al. 2023). Therefore, based on morphology and phylogenetic evidence, we establish this fungus as *Phyllosticta decaspermi* sp. nov.

##### ﻿ Phyllosticta morellae

Taxon classificationAnimaliaBotryosphaerialesPhyllostictaceae

M.Y. Zhang, Z.X. Zhang & X.G. Zhang sp. nov.

5F415251-4637-588D-AD74-F72F43330E40

857223

###### Etymology.

The specific epithet “*morellae*” refers to the host plant *Morella rubra*.

###### Type.

CHINA • Hainan Province, Ledong Li Autonomous County, Jianfengling National Forest Park, on diseased leaves of *Morella rubra* Lour., 14 October 2023, M.Y. Zhang(holotype HSAUP7509–5), ex-type living culture CGMCC3.28669.

###### Description.

**Figure 5.**
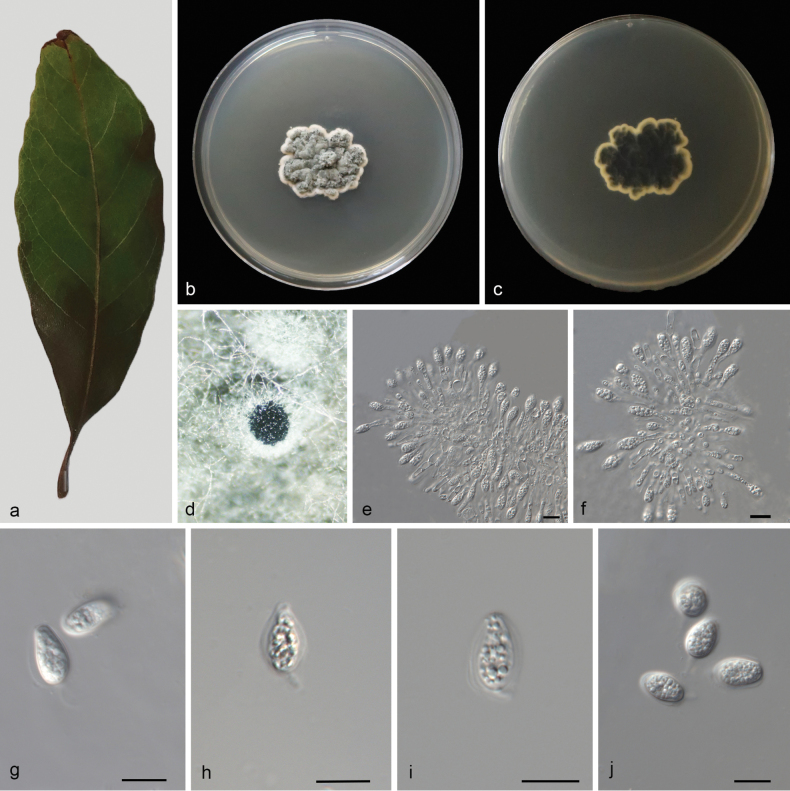
*Phyllosticta morellae* (CGMCC3.28669) **a** diseased leaf of *Morella rubra***b, c** colonies (left-above, right-reverse) after 7 d on PDA**d** conidiomata **e, f** conidiogenous cells with conidia **g–j** conidia. Scale bars: 10 μm (**e–j**).

Leaf endogenic and associated with leaves of *Morella rubra* Lour. Asexual morph: ***Conidiomata*** pycnidial, mostly aggregated in clusters, black, erumpent, globose to clavate or elongated with necks, in PDA culture exuding colourless to opaque conidial masses within 12 days or longer. ***Conidiophores*** indistinct, often reduced to conidiogenous cells. ***Conidiogenous cells*** 9.5–19 × 2–3 μm, subcylindrical, hyaline, smooth, proliferating several times percurrently near apex. ***Conidia*** 8.5–13.5 × 6–9 μm, solitary, hyaline, aseptate, thin and smooth walled, coarsely guttulate, or with a single large central guttule, ovoid to irregularly ellipsoid, surrounded by a mucilaginous sheath. ***Sheath*** 1–1.5 μm thick, and bearing a hyaline apical mucoid appendage. ***Appendages*** 2.5–5.5× 1 μm, flexible, unbranched, tapering towards an acutely rounded tip. Sexual morph not observed, see Fig. 5.

###### Culture characteristics.

Colonies on PDA 26–32 mm in diameter after 7 d at 25 °C in darkness, with a growth rate of 3.7–4.7 mm/day, white and undulate at edge, grey-green in center in obverse and reverse.

###### Additional specimen examined.

CHINA • Hainan Province, Ledong Li Autonomous County, Jianfengling National Forest Park, on diseased leaves of *Morella rubra* Lour., 14October 2023, M.Y. Zhang (HSAUP7515–2), living culture SAUCC7515–2.

###### Notes.

Two isolates from leaf spots of *Morella rubra* phylogeneticallyclustered into a well-supported clade (1.00/100), which is closely related to *P. decaspermi* (CGMCC3.28667), *Phyllosticta guangdongensis* (CFCC 58144) and *P. mangiferae* (IMI260.576). However, *P. morellae* differs from *P. decaspermi* by 114 nucleotides (6/559 in ITS, 7/743 in LSU,84/324 in *tef1*, 5/196 in *act*, and 12/715 in *gpdh*), from *P. guangdongensis* by 36 nucleotides (11/480 in ITS, 6/743 in LSU, 13/219 in *tef1*, 3/212 in *act*, and 3/594 in *gpdh*), and from *P. mangiferae* by 40 nucleotides (5/518 in ITS, 5/743 in LSU, 7/219 in *tef1*, 3/212 in *act*, and 20/621 in *gpdh*). In morphology, they are distinguished by different hosts (*Morella rubra* vs. *Decaspermum montanum* vs. *Viburnum odoratissimum* vs. *Mangifera indica*) and narrower conidia in *Phyllosticta decaspermi* than *P. guangdongensis*, *P. mangiferae* and *P. morellae* (8.5–13.5 × 6–9 μm vs. 10.2–13.4 × 5.6–6.8 μm vs. 10–14 × 6–8 μm vs. 10.0–12.0 × 6.0–7.0 μm) (Jeewon and Hyde 2016; Wang et al. 2023). Therefore, based on morphology and phylogenetic evidence, we establish this fungus as *Phyllosticta morellae* sp. nov.

#### ﻿*Phyllosticta concentrica* species complex

Based on molecular analysis and morphological characteristics, the *Phyllosticta concentrica* species complex comprises 34 species: *P. anhuiensis*, *P. aspidistricola*, *P. aucubae-japonicae*, *P. bifrenariae*, *P. catimbauensis*, *P. citriasiana*, *P. citribrasiliensis*, *P. citricarpa*, *P. citrichinensis*, *P. citri-maxima*, *P. clematidea*, *P. concentrica*, *P. cussonia*, *P. elliptica*, *P. elongata*, *P. ericarum*, *P. fujianensis*, *P. gardeniicola*, *P. gwangjuensis*, *P. hostae*, *P. hymenocallidicola*, *P. hypoglossi*, *P. iridigena*, *P. kerriae*, *P. kobus*, *P. ophiopogonis*, *P. paracitricarpa*, *P. pilospora*, *P. pittosporicola*, *P. rhododendri*, *P. speewahensis*, *P. turpiniae*, *P. westeae*, and *P. wuzhishanensis*.

##### ﻿ Phyllosticta clematidea

Taxon classificationAnimaliaBotryosphaerialesPhyllostictaceae

M.Y. Zhang, Z.X. Zhang & X.G. Zhang sp. nov.

B3F78E46-827B-52F6-99D7-F91FEA1DA223

859437

###### Etymology.

The specific epithet “*clematidea*” refers to the host plant *Clematis vitalba*.

###### Type.

CHINA • Yunnan Province, Yuxi City, Bailongtan Park, on diseased leaves of *Clematis vitalba*L., 12 May 2024, M.Y. Zhang (holotype HSAUP8121–1), ex-type living culture CGMCC3.28671.

###### Description.

**Figure 6.**
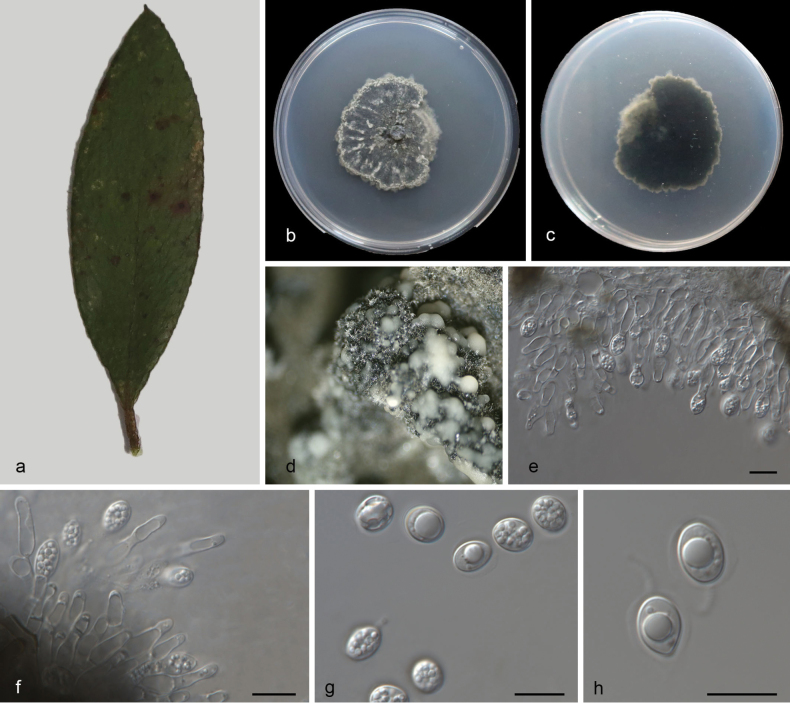
*Phyllosticta clematidea* (CGMCC3.28671) **a** diseased leaf of *Clematis vitalba***b, c** colonies (left-above, right-reverse) after 7 d on PDA**d** conidiomata **e, f** conidiogenous cells with conidia **g, h** conidia. Scale bars: 10 μm (**e–h**).

Leaf endogenic and associated with leaves of *Clematis vitalba*. Asexual morph: ***Conidiomata*** pycnidial, mostly aggregated in clusters, black, erumpent, globose to clavate or elongated, exuding opaque to withe conidial masses, it exudes write conidial masses within 12 days or longer. ***Conidiophores*** indistinct, often reduced to conidiogenous cells. ***Conidiogenous cells*** 10–18 × 2–4.5 μm, subcylindrical, hyaline, smooth, proliferating several times percurrently near apex. ***Conidia*** 8–11.5 × 6–10 (5.5–7.5) μm,solitary, hyaline, aseptate, thin and smooth walled, coarsely guttulate, or with a single large central guttule, ovoid to irregularly ellipsoid, surrounded by a mucilaginous sheath. ***Sheath*** 1–2 μm thick, and bearing a hyaline apical mucoid appendage. ***Appendages*** 2.5–6 × 1 μm, flexible, unbranched, tapering towards an acutely rounded tip. Sexual morph not observed, see Fig. 6.

###### Culture characteristics.

Colonies on PDA38–43 mm in diameter after 7 d at 25 °C in darkness, with a growth rate of 5.4–6.1 mm/day, undulate at edge, grey white to black in obverse and reverse.

###### Additional specimen examined.

CHINA • Yunnan Province, Yuxi City, Bailongtan Park, on diseased leaves of *Clematis vitalba* L., 12 May 2024, M.Y. Zhang (HSAUP8222–3), living culture SAUCC8222–3.

###### Notes.

Two isolates from leaf spots of *Clematis vitalba* phylogenetically clustered into a well-supported clade (1.00/100), which is closely related to *P. concentrica* (CPC 18842) and *P. kerriae* (MAFF 240047). However, *P. clematidea* differs from *P. concentrica* by 55 nucleotides (9/513 in ITS, 4/743 in LSU,17/235 in *tef1*, 18/220 in *act*, and 7/615 in *gpdh*) and from *P. kerriae* (LSU and *gpdh* sequences are available)by 37nucleotides (9/572 in ITS, 9/242 in *tef1*, and 19/227 in *act*). In morphology, they are distinguished by different hosts (*Clematis vitalba* vs. *Hedera* sp. vs. *Kerria japonica*) and smaller conidia in *P. clematidea* than *P. concentrica* and *P. kerriae* (8–11.5 × 6–10 (5.5–7.5) μm vs. (10–)11–13(–14) ×(6–)8(–9) μm vs. 9.5–12.5 × 6.0–7.5 μm) (Motohashi et al. 2008; Wikee et al. 2013). Therefore, based on morphology and phylogenetic evidence, we establish this fungus as *Phyllosticta clematidea* sp. nov.

##### ﻿ Phyllosticta pittosporicola

Taxon classificationAnimaliaBotryosphaerialesPhyllostictaceae

M.Y. Zhang, Z.X. Zhang & X.G. Zhang sp. nov.

06472C27-7AC7-54B5-96A0-8C01AA4EBF3C

857225

###### Etymology.

The specific epithet “*pittosporicola*” refers to the host plant *Pittosporum illicioides*.

###### Type.

CHINA • Yunnan Province, Fengming Mountain, Kunming City (ASL: 1926 m; 25.09°N, 102.76°E),on diseased leaves of *Pittosporum illicioides* Makino, 29 July 2023, M.Y. Zhang (holotype HSAUP5342–3), ex-type living culture CGMCC3.28666.

###### Description.

**Figure 7.**
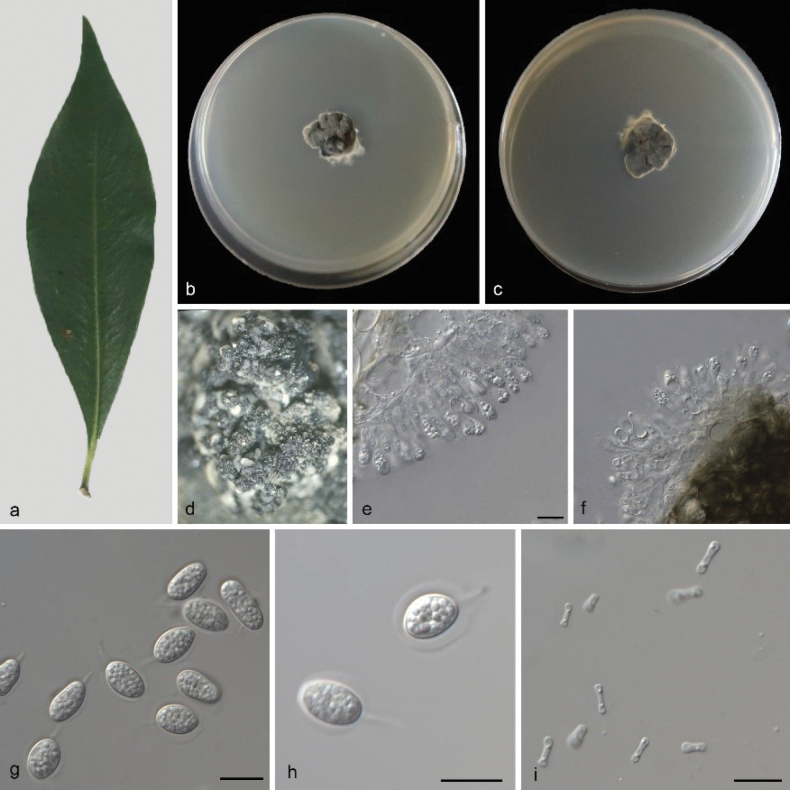
*Phyllosticta pittosporicola* (CGMCC3.28666) **a** diseased leaf of *Pittosporum illicioides***b, c** colonies (left-above, right-reverse) after 7 days on PDA**d** conidiomata **e, f** conidiogenous cells with conidia **g, h** conidia**i** spermatia. Scale bars: 10 μm (**e–i**).

Leaf endogenic and associated with leaves of *Pittosporum illicioides* Makino. Asexual morph: ***Conidiomata*** pycnidial, mostly aggregated in clusters, black, erumpent. In PDA culture exuding colourless to opaque conidial masses within 12 days or longer. ***Conidiophores*** indistinct, often reduced to conidiogenous cells. ***Conidiogenous cells*** 5.5–10 × 2–4 μm, subcylindrical, ampulliform, hyaline, smooth. ***Conidia*** 7–9 × 4–6 μm, hyaline, aseptate, thin and smooth walled, coarsely guttulate, ovoid, ampulliform, ellipsoidal to subglobose, enclosed in a thin mucoid sheath. ***Sheath*** 1–2 μm thick and bearing a hyaline, apical mucoid appendage. ***Appendages*** 4–7 × 1–2 μm, flexible, unbranched, tapering towards an acutely rounded tip. ***Spermatia*** 5–7.5 × 1–2.5 μm, occurring in conidioma with conidia, hyaline, smooth, guttulate to granular, bacilliform. Sexual morph not observed, see Fig. 7.

###### Culture characteristics.

Colonies on PDA 18–22 mm in diameter after 7 d at 25 °C in darkness, with a growth rate of 2.5–3.1 mm/day, undulate at edge, creamy white to black in obverse and reverse.

###### Additional specimen examined.

CHINA • Yunnan Province, Fengming Mountain, Kunming City (ASL: 1926 m; 25.09°N, 102.76°E),on dead leaves, 29July 2023, M.Y. Zhang (HSAUP5512–4), living culture SAUCC5512–4.

###### Notes.

Two isolates from leaf spots of *Pittosporum illicioides* phylogenetically clustered into a well-supported clade (1.00/100), which is closely related to *P. concentrica* (CPC 18842). However, *P. pittosporicola* differs from *P. elliptica* by 55 nucleotides (17/571 in ITS, 0/0 in LSU,18/406 in *tef1*, 18/220 in *act*, and 7/615 in *gpdh*). In morphology, they are distinguished by different hosts (*Clematis vitalba* vs. *Hedera* sp. vs. *Kerria japonica*) and shorter conidia in *Phyllosticta clematidea* than *P. concentrica* and *P. kerriae* (7–9 × 4–6 μm vs. (10–)11–13(–14) ×(6–)8(–9) μm vs. 9.5–12.5 × 6.0–7.5 μm) (Motohashi et al. 2008; Wikee et al. 2013). Therefore, based on morphology and phylogenetic evidence, we establish this fungus as *Phyllosticta pittosporicola* sp. nov.

#### ﻿*Phyllosticta cruenta* species complex

Based on molecular analysis and morphological characteristics, the *Phyllosticta cruenta* species complex comprises 22 species:*P. abieticola*, *P. cornicola*, *P. cruenta*, *P. cryptomeriae*, *P. foliorum*, *P. gaultheriae*, *P. hakeicola*, *P. hamamelidis*, *P. hubeiensis*, *P. illicii*, *P. leucothoicola*, *P. ligustricola*, *P. minima*, *P. neopyrolae*, *P. pachysandricola*, *P. paxistimae*, *P. podocarpicola*, *P. pyrolae*, *P. rubra*, *P. sphaeropsoidea*, *P. telopeae*, and *P. yuccae*.

#### ﻿*Phyllosticta owaniana* species complex

Based on molecular analysis and morphological characteristics, the *Phyllosticta owaniana* species complex comprises 6 species:*P. austroafricana*, *P. carissicola*, *P. hagahagaensis*, *P. owaniana*, *P. podocarpi*, and *P. pseudotsugae*.

#### ﻿*Phyllosticta rhodorae* species complex

Based on molecular analysis and morphological characteristics, the *Phyllosticta rhodorae* species complex comprises 3 species:*P. jiangxiensis*, *P. mimusopisicola*, and *P. rhodorae*.

#### ﻿*Phyllosticta vaccinii* species complex

Based on molecular analysis and morphological characteristics, the *Phyllosticta vaccinii* species complex comprises 2 species: *P. vaccinii* and *P. vacciniicola*.

## ﻿Discussion

In modern fungal taxonomy, integrating molecular data with morphological traits is essential, as traditional classification systems show increasing limitations. Mycologists now rely on divergence time estimates and phylogenomic data to clarify taxonomic boundaries (Baayen et al. 2002; Glienke et al. 2011; Wikee et al. 2013a, 2013b; Crous et al. 2014, 2015, 2016, 2017, 2018, 2019, 2021;Zhou et al. 2015; Guarnaccia et al. 2017; Lin et al. 2017; Hattori et al. 2020; Norphanphoun et al. 2020; Cravero et al. 2022; Li et al. 2022; Zhang et al. 2023; Mukhopadhyay et al. 2025). For *Phyllosticta*, historical identification depended on morphology and host associations, but overlapping traits hindered species delimitation (Norphanphoun et al. 2020). Molecular phylogenetics has improved species recognition, with ITS serving as a primary genus-level marker, while additional loci (LSU, *tef1*, *act*, and*gpdh*) are needed for species-level resolution (Jayawardena et al. 2019; Norphanphoun et al. 2020). To date, six major species complexes (100 accepted species) are recognized, including *P. capitalensis* (34 spp.), *P. concentrica* (34 spp.), *P. cruenta* (22 spp.), *P. owaniana* (6 spp.), *P. rhodorae* (3 sp.), and *P. vaccinii* (2 spp.).

In this study, four *Phyllosticta* isolates, recovered from four host genera (*Clematis vitalba*, *Decaspermum montanum*, *Pittosporum illicioides*, and *Morella rubra*), were described and illustrated through an integrative approach combining multi-locus phylogenetic analysis and morphology. Based on these analyses, four new species are proposed: *Phyllosticta decaspermi* sp. nov. (associated with *Decaspermum montanum* and dead leaves), *P. morellae* sp. nov. (from *Morella rubra*), *P. clematidea* sp. nov. (isolated from *Clematis vitalba*), and *P. pittosporicola* sp. nov. (obtained from *Pittosporum illicioides* and dead leaves). These newly described taxa are phylogenetically assigned to the *P. capitalensis* and *P. concentrica* species complexes, further expanding the known diversity within these clades. The USDA Fungal Database, a comprehensive repository of fungal-host associations, currently documents over 7,500 records of *Phyllosticta* species linked to plant hosts (excluding synonyms) (Okane et al. 2001, 2003; Baayen et al. 2002; Glienke et al. 2011; Wikee et al. 2013b; Wu et al. 2014; Zhang et al. 2015; Jayawardena et al. 2019; Tran et al. 2019; Hattori et al. 2020; Norphanphoun et al. 2020; Zhang et al. 2022; Gomdola et al. 2024; Jiang et al. 2024). This extensive dataset underscores the genus’s broad host range and ecological versatility. Among these, *P. capitalensis* stands out as a globally distributed generalist: it functions as both a common endophyte and a weak plant pathogen, with over 400 host-associated records in the database, reflecting its ability to colonize diverse plant species across various ecosystems (Glienke-Blanco et al. 2002; Silva and Pereira 2007; Silva et al. 2008; Wikee et al. 2013a). The addition of the four new species in this study contributes to this growing body of knowledge, highlighting the ongoing discovery of hidden diversity within *Phyllosticta*.

The evolutionary dynamics and functional differentiation of *Phyllosticta* species, revealed by our phylogenomic tree, gene family analysis, and KEGG metabolic pathway analysis, align with well-documented patterns in fungal genomic studies. For phylogenomic resolution, the strongly supported clades in *Phyllosticta* (e.g., the *P. concentrica* and *P. capitalensis* species complexes) mirror the robust phylogenetic relationships inferred for the Terminal *Fusarium* Clade using 1,049 single-copy orthologs (Lizcano Salas et al. 2024). This consistency highlights the utility of phylogenomics—specifically, single-copy ortholog datasets—for resolving ambiguous species boundaries in filamentous fungi, a challenge also noted in *Phyllosticta* due to morphological overlap among species.

Metabolic pathway specialization provides additional insights into *Phyllosticta*’s functional divergence, with patterns supported by both our data and relevant fungal studies. For *P. saprophytica*, enhanced activity of the “Protein processing in endoplasmic reticulum” pathway matches its saprophytic lifestyle. This pathway mediates the folding and modification of secreted hydrolytic enzymes (e.g., cellulases and ligninases) that are key to decomposing complex organic matter in dead plant tissues. This mechanism is supported by findings in *Trichoderma reesei* (Yao et al. 2022). For *P. rhododendri*, elevated “Amino sugar and nucleotide sugar metabolism” correlates with its host-associated (endophytic/weakly pathogenic) lifestyle. This pathway produces precursors for synthesizing chitin and glycoproteins, which are key components of fungal cell walls and play roles in evading host immune recognition (e.g., in the plant-associated fungus *Colletotrichum gloeosporioides*; Chen et al. 2023). These metabolic adaptations parallel findings in *Bipolaris* species, where pathway-specific activity directly correlates with ecological niches (e.g., oxidative phosphorylation linked to pathogenicity; Song et al. 2024).

Notably, the morphological overlap among *Phyllosticta* species reinforces the necessity of multi-locus phylogeny (or phylogenomics) for accurate species delimitation—an argument similarly emphasized in studies on *Diaporthe* (Dissanayake et al. 2024; Zhang et al. 2025). In summary, *Phyllosticta*’s evolutionary trajectory is shaped by nuclear genomic divergence, as evidenced by phylogenomic clade structure and gene family dynamics, which drive metabolic specialization. These processes enable adaptation to diverse hosts and ecological niches, consistent with conserved patterns in filamentous fungal evolution (e.g., *Fusarium* and *Bipolaris*; Lizcano Salas et al. 2024; Song et al. 2024).

## Supplementary Material

XML Treatment for Phyllosticta decaspermi

XML Treatment for Phyllosticta morellae

XML Treatment for Phyllosticta clematidea

XML Treatment for Phyllosticta pittosporicola

## ﻿Additional information

### Use of AI

No use of AI was reported.

### Adherence to national and international regulations

All the fungal strains used in this study have been legally obtained, respecting the Convention on Biological Diversity (Rio Convention).

### Author ORCIDs

Meng-Yuan Zhang https://orcid.org/0009-0002-3068-7491

Zhao-Xue Zhang https://orcid.org/0000-0002-4824-9716

Ya-Ling Wang https://orcid.org/0009-0009-4029-2734

Xiu-Guo Zhang https://orcid.org/0000-0001-9733-8494

### Data availability

All of the data that support the findings of this study are available in the main text or Supplementary Information.

## Supplementary materials

Supplementary material 1 Species and GenBank accession numbers of DNA sequences used in this study Data type docx https://binary.pensoft.net/file/1444179This dataset is made available under the Open Database License (http://opendatacommons.org/licenses/odbl/1.0/). The Open Database License (ODbL) is a license agreement intended to allow users to freely share, modify, and use this Dataset while maintaining this same freedom for others, provided that the original source and author(s) are credited.Meng-Yuan Zhang, Zhao-Xue Zhang, Ya-Ling Wang, Xiu-Guo Zhang, Zhuang Li

Supplementary material 2 The sequence of phylogenetic analysis Data type txt https://binary.pensoft.net/file/1444180This dataset is made available under the Open Database License (http://opendatacommons.org/licenses/odbl/1.0/). The Open Database License (ODbL) is a license agreement intended to allow users to freely share, modify, and use this Dataset while maintaining this same freedom for others, provided that the original source and author(s) are credited.Meng-Yuan Zhang, Zhao-Xue Zhang, Ya-Ling Wang, Xiu-Guo Zhang, Zhuang Li

Supplementary material 3 Single phylogenetic analysis Data type pdf https://binary.pensoft.net/file/1444181This dataset is made available under the Open Database License (http://opendatacommons.org/licenses/odbl/1.0/). The Open Database License (ODbL) is a license agreement intended to allow users to freely share, modify, and use this Dataset while maintaining this same freedom for others, provided that the original source and author(s) are credited.Meng-Yuan Zhang, Zhao-Xue Zhang, Ya-Ling Wang, Xiu-Guo Zhang, Zhuang Li
