# Supplementary figures and images for: ﻿Molecular and morphological characterization of four new Phyllosticta species (Botryosphaeriales, Phyllostictaceae): Genomic insights into evolutionary dynamics and metabolic adaptation
Source: IMA Fungus. 2025 Oct 20;16:e168055. doi: 10.3897/imafungus.16.168055 (PMC12559956; doi:10.3897/imafungus.16.168055)

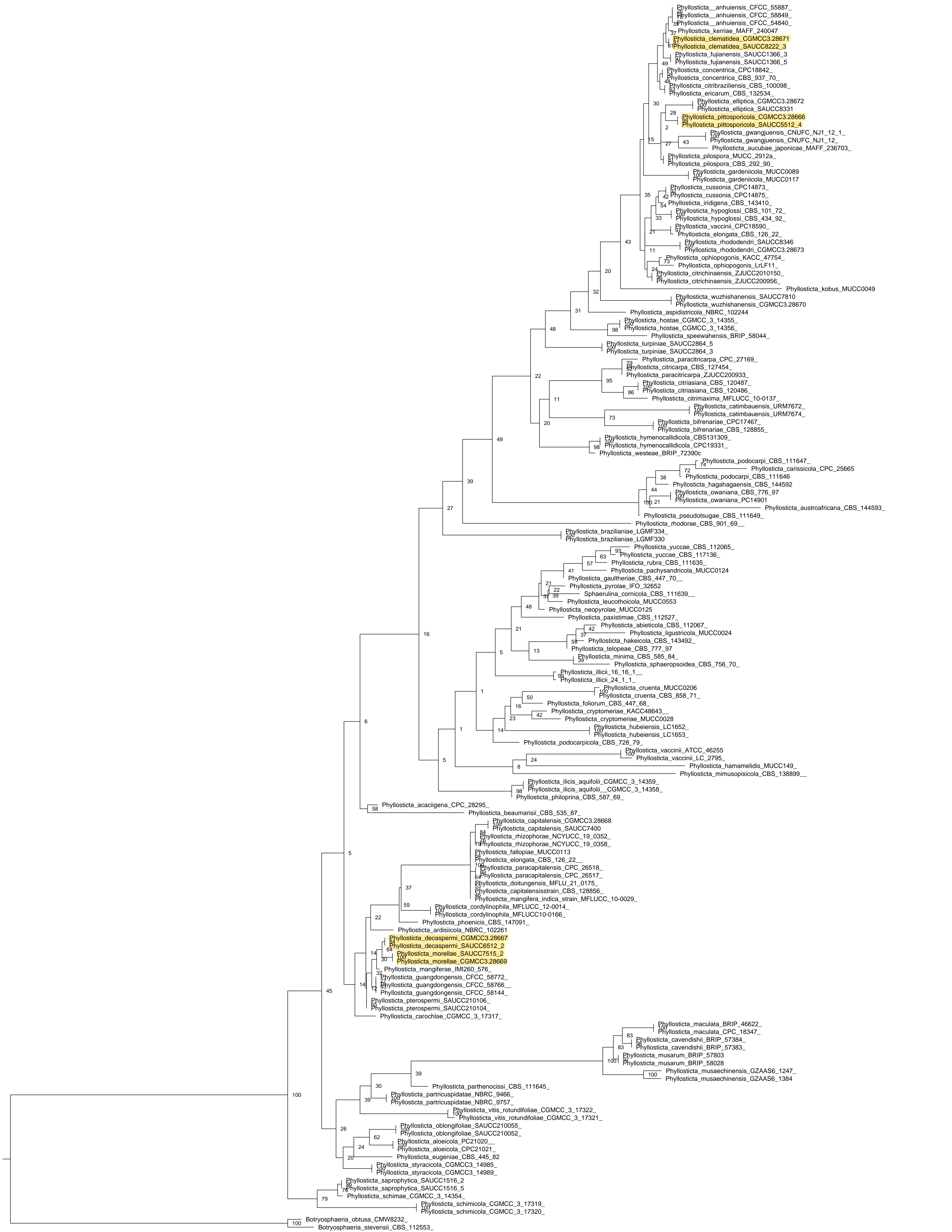

Supplement: Supplementary material 3 — Single phylogenetic analysis [file imafungus-16-e168055-s003.pdf]
